# Crystal structure of *Anoxybacillus* α-amylase provides insights into maltose binding of a new glycosyl hydrolase subclass

**DOI:** 10.1038/srep23126

**Published:** 2016-03-15

**Authors:** Kian Piaw Chai, Noor Farhan Binti Othman, Aik-Hong Teh, Kok Lian Ho, Kok-Gan Chan, Mohd Shahir Shamsir, Kian Mau Goh, Chyan Leong Ng

**Affiliations:** 1 Universiti Teknologi Malaysia, Faculty of Biosciences and Medical Engineering, 81310 Skudai, Johor, Malaysia; 2 Universiti Kebangsaan Malaysia, Institute of Systems Biology, 43600 UKM Bangi, Selangor, Malaysia; 3 Universiti Sains Malaysia, Centre for Chemical Biology, 11800 Penang, Malaysia; 4 Universiti Putra Malaysia, Department of Pathology, Faculty of Medicine and Health Sciences, 43400 Serdang, Selangor, Malaysia; 5 University of Malaya, Division of Genetics and Molecular Biology, Institute of Biological Sciences, Faculty of Science, 50603 Kuala Lumpur, Malaysia

## Abstract

A new subfamily of glycosyl hydrolase family GH13 was recently proposed for α-amylases from *Anoxybacillus* species (ASKA and ADTA), *Geobacillus thermoleovorans* (GTA, Pizzo, and GtamyII), *Bacillus aquimaris* (BaqA), and 95 other putative protein homologues. To understand this new GH13 subfamily, we report crystal structures of truncated ASKA (TASKA). ASKA is a thermostable enzyme capable of producing high levels of maltose. Unlike GTA, biochemical analysis showed that Ca^2+^ ion supplementation enhances the catalytic activities of ASKA and TASKA. The crystal structures reveal the presence of four Ca^2+^ ion binding sites, with three of these binding sites are highly conserved among *Anoxybacillus* α-amylases. This work provides structural insights into this new GH13 subfamily both in the apo form and in complex with maltose. Furthermore, structural comparison of TASKA and GTA provides an overview of the conformational changes accompanying maltose binding at each subsite.

α-Amylases (EC 3.2.1.1) cleave α-1,4-glycosidic bonds of carbohydrates and oligosaccharides; Hence, these amylolytic enzymes are employed for industrial starch liquefaction and saccharification^1^. In living organisms, α-amylases are important in starch metabolism (KEGG map ec00500) for digesting carbohydrates to simpler sugars. α-Amylases belong to the glycoside hydrolase (GH) family, which contains nearly 28,000 protein sequences with various specificities^2^,^3^. Approximately 83% of these sequences belong to the 40 curator-based subfamilies (as of Nov 2015) in the GH13 family^3^,^4^,^5^, and 11 subfamilies exhibit α-amylase specificity: GH13_1 (fungi), GH13_5 (bacterial liquefying enzymes), GH13_6 (plants), GH13_7 (archaea), GH13_15 (insects), GH13_24 (animals), GH13_27, GH13_28 (bacterial saccharifying enzymes), GH13_32, GH13_36 (intermediary), and GH13_37 (marine bacteria)^2^. GH13 family enzymes have three structural domains: (i) domain A, which forms an N-terminal (β/α)_8_ fold (TIM barrel) that functions as the catalytic domain; (ii) domain B, which is essential for substrate binding; and (iii) domain C, which forms a β-sandwich designated as a putative saccharide binding site^6^.

Recently, a new α-amylase GH13 subfamily was proposed to encompass enzymes found in thermophilic *Anoxybacillus* and *Geobacillus* species and in a halophilic *Bacillus* species^7^. The earliest two representatives of this subfamily, ASKA and ADTA from *Anoxybacillus* sp. SK3-4 and DT3-1, respectively, produce high levels of maltose upon reacting with starch^8^. In our earlier study^9^, we suggested that ASKA and amylopullulanase anchor to the cells of *Anoxybacillus* sp. SK3-4, which is an important adaptation to the native hot spring environment of *Anoxybacillus* sp. SK3-4^10^. Both enzymes work synergistically to hydrolyse starch to glucose, maltose, and maltodextrins^9^. Phylogenetic analysis suggests that ASKA and ADTA cluster with the *Bacillus aquimaris* α-amylase BaqA^11^ and the *G. thermoleovorans* α-amylases Pizzo^12^, GTA^13^, and GtamyII^14^, which exhibit similar conserved sequence regions (CSRs)^7^. Further phylogenetic analysis of 95 homologous sequences to ASKA indeed indicated that ASKA belongs to a novel GH13 subfamily. Members of this subfamily are characterized by a pair of tryptophan residues between CSR-V and CSR-II, the five-residue LPDIx signature in CSR-V, and a long C-terminal region containing five conserved aromatic residues^3^.

The crystal structure of GTA (PDB ID: 4E2O) provided the first insight into the overall structure, Ca^2+^ binding sites, and substrate binding subsites of this new GH13 subfamily of α-amylases^13^. Since, GTA is the only structure available in this new subclass of GH, elucidation of homologous structures is expected to increase understanding of the unique GH13 subfamily. Here, we present the first α-amylase structure from *Anoxybacillus* in the unaffiliated GH13 subfamily.

## Results

### Structures of TASKA-Apo and TASKA-ligand complexes

**Figure 1:**
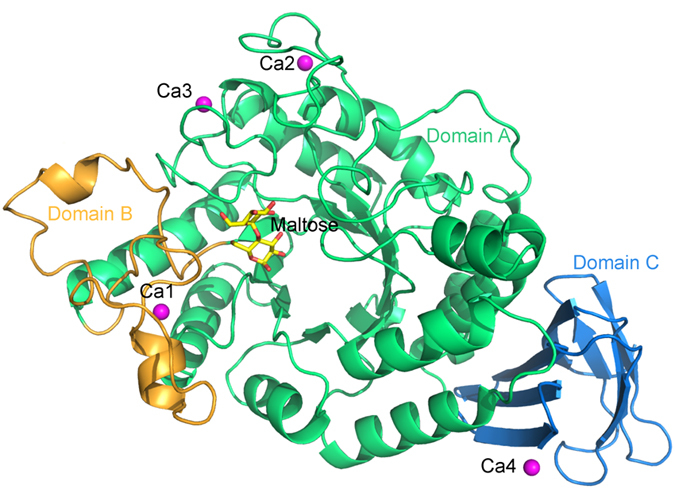
Overall structure of truncated α-amylase GH13 subfamily of *Anoxybacillus* species (TASKA). Domains A, B, and C are shown in green, orange, and blue, respectively. The calcium ions are shown in magenta.

To improve the efficiency of recombinant protein purification, we truncated 23 and 27 residues from the N- and C-termini of ASKA, respectively. Consequently, the residue numbering in this report is according to the position in TASKA unless otherwise specified. Crystal structures of the apo form (TASKA-Apo; PDB ID: 5A2A), the maltose-bound complex (TASKA-M; PDB ID: 5A2B), and the maltotriose-bound complex (TASKA-T; PDB ID: 5A2C) were determined to 1.85–1.95 Å resolution. All the crystals belonged to space group P2_1_2_1_2_1_, with one monomer in the asymmetric unit. The overall structure resembles previously solved structures of GH13 α-amylases^13^,^15^ and consists of three domains^16^: catalytic domain A containing the active site within its TIM barrel fold (residues 26–139, 187–393), domain B (residues 140–186), and domain C with an all-β fold (residues 394–475) (Fig. 1). The 3D structural alignment using the DALI database^17^ reveals that the overall structure of TASKA-Apo is similar to those of GTA, *Aspergillus niger* α-amylase or TAKA-amylase, *G. stearothermophilus* maltogenic α-amylase Novamyl, *G. stearothermophilus* neopullulanase, *B. circulans* cyclodextrin glycosyltransferase (CGTase), and other GHs (Table S1).

Despite its high overall structural similarity to the first three domains of Novamyl (PDB ID: 1QHO), we did not identify maltose bound to the surface of TASKA domain C, which contains a saccharide binding site in the *Bacillus amyloliquefaciens* α-amylase, Novamyl, and *B. circulans* CGTase structures previously reported^18^,^19^,^20^. Notably, TASKA did not have an amino acid sequence similar to these counterparts. Members of the GH13 subfamily, including Pizzo, GtamyII, and BaqA, can degrade raw starch; however, the putative binding sites suspected in domain C have yet to be identified^14^.

### Ca^2+^ ion binding to TASKA

Previous biochemical studies showed that Ca^2+^ ion supplementation in enzymatic reactions did not enhance the catalytic activity of GTA but improved those of ASKA and TASKA. Additional Ca^2+^ binding sites may contribute to the stimulatory effect of Ca^2+^ observed in ASKA and TASKA but not in GTA^7^,^8^,^13^. Although GTA contains only two Ca^2+^ binding sites^13^ and shows high sequence identity to TASKA, we identified four Ca^2+^ ions (Ca1–4 in subsequent sections) in the TASKA-Apo, TASKA-M, and TASKA-T structures (Fig. 1). The four Ca^2+^ ions in the TASKA structures were assigned according to the electron density level and coordination stereochemistry of Ca^2+^ binding. It is known that Ca^2+^ typically forms octahedral coordination geometry with a coordination number (CN) of 5–8 to complete the coordination sphere and bonding distance of ~2.4 Å. The peptide residues that commonly coordinate Ca^2+^ are Asp, Asn, and Glu^21^.

**Figure 2:**
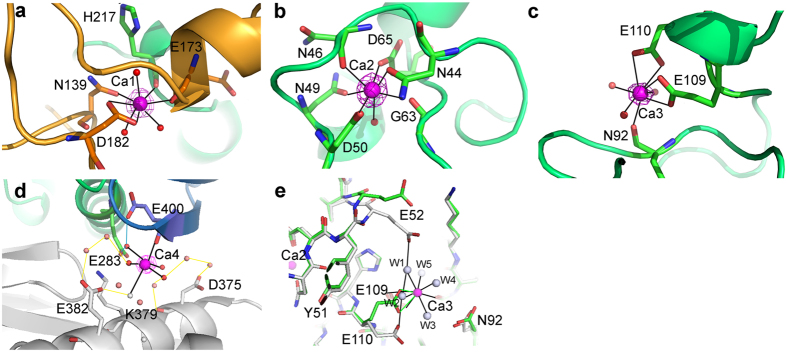
Calcium ion binding sites in *Anoxybacillus* α-amylase family GH13. Ca^2+^ ions are shown in magenta, and water molecules that directly interact with Ca^2+^ ions are shown in red. The Fo-Fc omit maps for the Ca^2+^ ions are contoured at 8.0σ. The interactions between Ca^2+^ ions, water molecules, and amino acid residues are shown with black lines. (**a**) Ca1 bridges domains A and B of TASKA by interacting with three water molecules and four conserved residues. (**b**) Ca2 interacts with universally conserved residues N49, D50, G63, and D65 and *Anoxybacillus* conserved residues N44 and N46. (**c**) Ca3 interacts with the side chains of *Anoxybacillu*s conserved residues E109 and E110 and the main chain of N92. (**d**) Ca4 interacts with conserved residue E400 and five water molecules. A Ca4-binding water molecule interacts with *Anoxybacillus* conserved residues E283 and E400 (blue lines). The hydrogen bond networks between TASKA and symmetry-related molecules in the crystal are shown in yellow. (**e**) The side chain of conserved residue E110 shows a conformational change in TASKA-M (grey) compared to TASKA-Apo and TASKA-T (green). Two water molecules (W1 and W2) exclusively found in TASKA-M interact directly with Ca3. W1 and W2 bridge Ca3 and residues E52 and E110, respectively. Interestingly, the backbone of E52 is flipped to adopt the bridge with Ca3. The cause of these conformational changes is unknown.

Ca1 is coordinated by the conserved residues N139 and D182, the main-chain oxygen atoms of H217 and E173, and three water molecules (Fig. 2a). Ca2 is coordinated by the conserved residues N44, N49, D50, and D65; the main-chain oxygen atoms of N46 and G63; and a water molecule (Fig. 2b). Ca3 is coordinated by the side chains of E109 and E110, the main-chain oxygen atom of N92, and three water molecules in TASKA-Apo and TASKA-T, whereas this Ca^2+^ ion is coordinated by residues E109 and N92 and five water molecules in TASKA-M (Fig. 2c). Despite lacking Ca^2+^-interacting residues (Asp, Asn, and Glu), a Ca4 binding site with CN 6–7 and ion-oxygen bonding distances of ~2.5 Å was also assigned in TASKA structures. Notably, although both Cl^−^ and Ca^2+^ ions are present in the crystallization buffer and have the same electron numbers, we ruled out Cl^−^ as the bound ligand since the site does not obey the stereochemical criteria of Cl^−^ binding^22^. Ca4 was coordinated by the main-chain oxygen atom of E400 and five water molecules (TASKA-Apo) or six water molecules (TASKA-M and TASKA-T), with one forming hydrogen bond interactions with conserved residues E283 and E400 (Fig. 2d).

Comparison of the Ca^2+^ ion binding sites of all three TASKA structures reveals that the side-chain of residue E110, which interacts with Ca3, underwent a conformational change in the TASKA-M structure relative to TASKA-Apo and TASKA-T. Two water molecules exclusively found in TASKA-M interact directly with Ca3 and bridge it with E52 and E110. Notably, E52 is not involved directly or indirectly in Ca3 binding in TASKA-Apo and TASKA-T, whereas a conformational change flips the main-chain peptide of Y51 to allow the side chain of E52 to interact with Ca3 in TASKA-M. The cause of this conformational change remains unclear (Fig. 2e).

As four Ca^2+^ ion binding sites are rarely reported, we used comparative sequence analysis to determine whether these Ca^2+^-interacting residues are conserved in other α-amylases from *Anoxybacillus* spp., *Geobacillus* spp., and *Bacillus* spp. Sequence alignment suggests that all the residues that interact with the four Ca^2+^ ions are well conserved in most α-amylases from *Anoxybacillus* spp. except *A. tepidamans*, suggesting that *Anoxybacillus* α-amylases may generally have four Ca^2+^ ion binding sites. The α-amylase of *A. tepidamans* contains conserved Ca^2+^ binding residues only for Ca1, Ca2, and Ca4. (Fig. S1). Phylogenetic analyses of 16S rRNA and α-amylase genes further support *A. tepidamans*, which was initially reported as *Geobacillus tepidamans*, as an outlier of *Anoxybacillus* spp. (Fig. S2a,b)^23^. The 16S rRNA sequence of *A. tepidamans* is phylogenetically positioned at the base of the *Anoxybacillus* cluster and represents the closest taxon between the *Anoxybacillus* species and the16S rRNA of the *Geobacillus* spp. cluster. Therefore, *A. tepidamans* is likely a more divergent representative of the GH13 subgroup of the *Anoxybacillus* cluster (Fig. S2a,b).

To further confirm the endogenous calcium content of TASKA, we performed isothermal titration calorimetry (ITC). Titration of TASKA with 1 mM EDTA identified only two Ca^2+^ ion binding sites in TASKA (Fig. S3), presumably representing the Ca^2+^ ions at the conserved Ca1 and Ca2 binding sites. However, there were technical constraints on ITC analysis when the Ca^2+^ ion concentration was increased to 5 mM or higher, hence limiting analysis under conditions mimicking those of crystallization.

### Structural comparison of TASKA-Apo and TASKA-ligand

**Figure 3:**
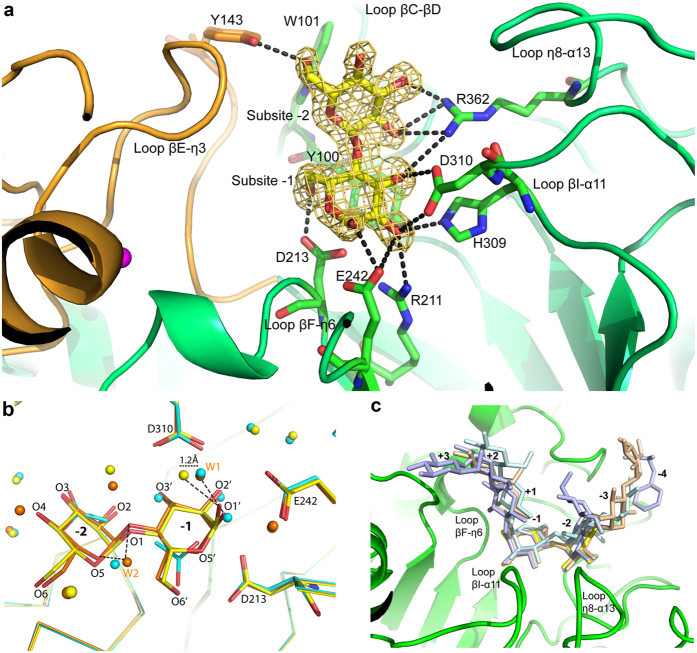
Structural analysis of maltose binding at subsites −1 and −2 of TASKA. (**a**) Maltose binding at the active sites of *Anoxybacillus* α-amylase family GH13. Hydrogen bonds are shown as dotted lines. The 2Fo-Fc maps for maltose are contoured at 1.0σ. (**b**) Superposition of TASKA-Apo (cyan), TASKA-M (orange), and TASKA-T (yellow) structures shows the conformational changes in the substrate binding sites. The binding site of TASKA-Apo contains solvent molecules, whereas those of TASKA-M and TASKA-T contain maltose at subsites −1 and −2 and show substantial differences, especially at O1′. Coordination of a water molecule (W1) deviates 1.2 Å in TASKA-T, and another water molecule (W2) hydrogen bonded with the O1 and O5 atoms of maltose in TASKA-M was absent in TASKA-T. (**c**) Superposition of TASKA-T (yellow) and α-amylase complexes including GTA-acarbose (PDB ID: 4E2O) (brown), *Bacillus stearothermophilus* maltogenic α-amylase Novamyl (PDB ID: 1QHO) (light blue), and pig pancreatic α-amylase (PDB ID: 1PPI) (pale cyan) shows that the disaccharide coordination of subsites −1 and −2 is highly conserved.

Structural superposition of TASKA-Apo with TASKA-M and TASKA-T using protein backbone C_α_ atoms shows low RMSD values of 0.32 and 0.20 Å, respectively, indicating that the overall structure of TASKA does not undergo significant conformational changes upon maltose binding. In the absence of a substrate or product, the structure of TASKA-Apo reveals that the active site of the enzyme is occupied by water and solvent molecules, whereas the structures of TASKA-M and TASKA-T show maltose bound to substrate-interacting subsites −1 and −2 of the substrate binding site (Fig. 3a) with each subsite able to interact with one glucose subunit of maltose^24^. These interactions are similar to the binding in GTA^13^. The structure of TASKA-T contains electron density representing a maltose molecule at the same subsites as in TASKA-M, suggesting that maltotriose was likely hydrolysed during crystallization. The hydrolytic activity for maltotriose was confirmed in a separate study using an Ultra Performance Liquid Chromatography-Evaporating Light Scattering Detector (UPLC-ELSD), wherein glucose and maltose were produced following the hydrolysis of maltotriose (Fig. S4). Therefore, TASKA-T represents a similar enzyme-product complex structure to TASKA-M.

**Figure 4:**
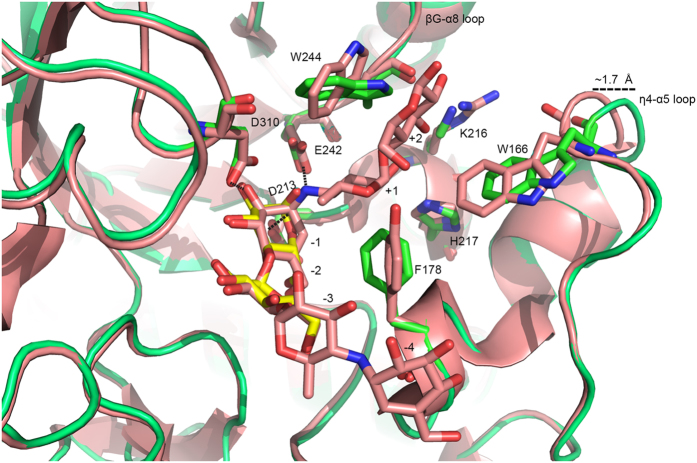
Superposition of the TASKA-maltose and GTA-acarbose structures shows the conserved maltose binding site at subsites −1 and −2. W244 and loop η4-α5 are proposed to undergo conformational changes upon substrate binding. The conserved Asp-Glu-Asp catalytic triad (D213, E242, and D310) is shown as sticks. TASKA is shown in green, and GTA is shown in salmon. Maltose from TASKA-maltose is shown in yellow.

In the TASKA-M and TASKA-T complex structures (Fig. 3b), the O1′, O2′, and O3′ atoms of maltose were positioned identically to three of the water molecules in the TASKA-Apo structure, and the O5′, O6′, and O3 of maltose were coordinated similarly to the oxygen atoms of the acetate ions. The water and acetate molecules interact with the conserved maltose-binding residues R211, D310, D213, and R362. In both the TASKA-M and TASKA-T structures, the maltose coordinates the βC-βD, βE-η3, βF-η6, βI-α11, and η8-α13 loops near the entry site of the TIM barrel and forms hydrogen bonds with the side chains of the Asp-Glu-Asp catalytic triad residues (D213, E242, and D310) and W101, Y143, R211, H309, and R362 (Fig. 3a). The subsite −1 ring forms a π-π interaction with the side chain of conserved Y100, which is common to the GH13 family^25^. Structural comparisons revealed that maltose binding at subsites −1 and −2 of the TASKA active site is highly similar to that in GTA (PDB ID: 4E2O), *G. stearothermophilus* maltogenic α-amylase (PDB ID: 1QHO)^18^, *B. circulans* CGTase (PDB ID: 1EO5)^26^, and many other homologues in the DALI database (Fig. 4).

To provide a dynamic view of the conformational changes upon maltose binding to each subsite, we performed molecular dynamics (MD) simulations in the presence and absence of maltose. Trajectory analysis of the root-mean-square deviation (RMSD) plot (Fig. S5a) showed that TASKA-Apo and TASKA-T reached equilibrium states after 8 ns and 9 ns of simulation, respectively. The root-mean-square fluctuations (RMSF) plot of TASKA-T relative to TASKA-Apo suggested the presence of flexible regions (Fig. S5b) that η2-α2 and η8-α13 loops in close proximity to maltose binding residues (Fig. S5c). The residues that interact with subsites −1 and −2 were highly stable, with main chain C_α_ atoms undergoing conformational changes with RMSD of ~1–2 Å. The βG-α8 loop that moved ~6 Å and the η4-α5 loop (RMSD 2.6 Å) that may participate in subsite +2 coordination have been proposed to be important in accommodating subsite +2, as described in the discussion section. The η2-α2 loop was highly flexible (RMSD of ~6 Å); however, the physiological function of this loop remains to be investigated (Fig. S5c).

## Discussion

The α-amylases from *Anoxybacillus* species (ASKA and ADTA), *G. thermoleovorans* (GTA, Pizzo, and GtamyII), and *B. aquimaris* (BaqA) have been proposed as a novel subfamily of the α-amylase family^7^, which was recently supported by detailed phylogenetic analysis^3^. The GH13 subfamily uniquely exhibits (i) high maltose production; (ii) the ability to degrade raw starch; (iii) a long C-terminal sequence containing five conserved aromatic residues; (iv) dual tryptophan residues between CSR-V and CSR-II; and (v) a unique stretch of amino acids (LPDIx) in CSR-V^3^,^7^.

Recently, a structure of GTA complexed with an acarbose-derived pseudo-hexasaccharide was determined, providing the first structural insights into this new subfamily of GH13 α-amylases^13^. Previous biochemical analysis showed that addition of Ca^2+^ ions does not change the catalytic activity of GTA but enhances the activities of ASKA and TASKA^7^,^8^,^13^. Despite the 69% sequence similarity between the primary sequences of TASKA and GTA, the TASKA structure contains four well-ordered Ca^2+^ ion binding sites, whereas GTA has only two Ca^2+^ ion sites (with only one well-ordered in the structure). All the TASKA Ca^2+^ ions are located far from the catalytic site, suggesting that they may not be directly involved in catalysis, and analyses suggest that Ca1, Ca2, and Ca3 are likely to be important for structural stability. Ca1 and Ca2 occupy the two calcium-binding sites conserved among the members of GH13. Ca1 bridges domains A and B, and this interface is important for α-amylase and CGTase thermostability^27^,^28^,^29^,^30^. Ca1-interacting residues are sensitive to amino acid substitution, and mutagenesis studies are often deleterious^28^,^31^,^32^. Ca2 and Ca3 are positioned in domain A and may help stabilize the catalytic domain. Amino acid substitutions at residues adjacent to Ca2-interacting residues can increase the optimum temperature and exhibit calcium-independent behaviour^33^. Both Ca1 and Ca2 are coordinated similarly to those in GTA, and coordination of Ca1, Ca2, and Ca3 was previously found in the five domains of *G. stearothermophilus* α-amylase (PDB ID: 1QHO)^18^. Sequence alignment suggests that residue N46, which interacts with Ca2, and residues E109 and E110, which interact with Ca3, are conserved in almost all *Anoxybacillus* α-amylases except *A. tepidamans* (Fig. S1). In addition to the five previously defined unique features of this newly established GH13 subfamily, our current results suggest that Ca1, Ca2, and Ca3 binding sites and their interacting residues are a unique feature of *Anoxybacillus* α-amylases but are less conserved in GTA, Pizzo, GtamyII, and BaqA α-amylases from the *Geobacillus* and *Bacillus* genera.

Superposition of TASKA and GTA reveals that the Ca3 binding residue E110 is substituted with a proline residue (P114) in GTA. Furthermore, the substitution of P98 in GTA with K94 in TASKA weakens the hydrogen bond between the main chains of adjacent residues E93–W101 (3.15 Å) in TASKA relative to M97–W105 (2.75 Å) in GTA, hence decreasing TASKA’s structural stability. We hypothesize that the substitution of two GTA proline residues (P98 and P114) with K94 and E110, respectively, in TASKA may increase the flexibility of the loop region around Ca3, which may have been recruited for structure stabilization (Fig. 2c). Structural comparisons also reveal that the carboxylic group of E110, which directly interacts with Ca3 in TASKA-Apo and TASKA-T, adopts different side-chain rotamers and interacts with a Ca3-binding water molecule in the TASKA-M structure. In addition, another Ca3-binding water molecule found exclusively in TASKA-M forms a hydrogen bond with E52 that is accommodated by flipping the main chain of Y51 (Fig. 2e). We are unable to conclusively determine the underlying causes of the conformational changes observed in the TASKA-M crystal structure. The changes may have been triggered by changing the rotamer conformation of R322 in the crystal symmetry-related molecule, which is hydrogen bonded to the side chain of E50 in the TASKA-T structure. However, both TASKA-M and TASKA-T adopted highly similar crystal packing. TASKA-M was obtained through co-crystallization of TASKA and maltose, while TASKA-T represents TASKA in a post-maltotriose hydrolysis stage. It is also possible that the specific stages of TASKA-maltose complex formation would affect Ca3 coordination.

Ca4 was identified in TASKA-Apo, TASKA-T, and TASKA-M. Ca4 occupies a novel calcium-binding site on the surface of domain C and directly interacts with the oxygen atom of E400 and five (TASKA-Apo) or six water molecules (TASKA-T and TASKA-M), one of which is hydrogen bonded with the conversed E283 and E400 residues. Another two water molecules indirectly build a water network linking Ca4 to a symmetry-related TASKA molecule in the crystal; thus, given the lack of Ca^2+^-binding features^21^, the Ca4 site likely results from crystal packing (Fig. 2d). Nonetheless, it is worth noting that the homologous Novamyl and CGTase structures show interactions between maltose and the main-chain oxygen atom of I415, which is equivalent to TASKA E400^18^,^19^ (Fig. S6). However, the physiological relevance of Ca4 to *Anoxybacillus* α-amylases has yet to be confirmed.

The results of ITC experiments indicate that there are 1.6 calcium binding sites found in TASKA at the condition of 1 mM CaCl_2_, which we interpret as the conserved Ca1 and Ca2 binding sites in α-amylases. We note that 1 mM CaCl_2_ reaches saturation in TASKA. For comparison, the intracellular Ca^2+^ ion concentration in *E. coli* is ~100 nM^34^. While the ITC results for TASKA in 5 mM CaCl_2_ indicating the presence of 3.5 binding sites, the plot fitting curves were rather poor and likely not reliable. We are unable to validate the reliability of 3.5 Ca binding sites as proposed by ITC. This suggests that the experiment setup with Ca ion (5 mM) has reached the highly saturated condition that is not favorable to TASKA in the current ITC setup. Therefore, we were not able to conduct further ITC experiment that mimic crystallization environment, i.e. 0.1–0.2 M calcium acetate. Nonetheless, the ITC results do suggest that binding of Ca3 and Ca4 to respective pockets might be weaker than that of Ca1 and Ca2 and undetectable under the current experimental setup. We cannot eliminate the possibility that Ca3 and Ca4 binding in TASKA is induced by the high Ca^2+^ ion concentration in the crystallization setup. Thus, the Ca^2+^ binding pocket, particularly the one in domain C could be a general metal binding site.

To complement the understanding of enzyme conformations before and after substrate binding, we solved TASKA structures in the apo form and complexed with maltose or maltotriose. The structure of TASKA co-crystallized with maltotriose showed a well-ordered maltose in the substrate-binding site, suggesting that TASKA can hydrolyse maltotriose to maltose and glucose during the crystallization process. We hypothesize that TASKA-M mimics the enzyme-product complex, while TASKA-T is a snapshot structure of the enzyme after maltotriose hydrolysis (post-substrate-hydrolysis stage). Accordingly, TASKA-M and TASKA-T have slightly different maltose binding interactions.

Substrate binding at TASKA subsites −1 and −2 is highly similar to that of many α-amylases including Novamyl and pig pancreatic α-amylase (PDB ID: 1PPI)^35^, suggesting that the coordination of subsites −1 and −2 is well conserved (Fig. 3c). Superposition of the TASKA-Apo, TASKA-M, and TASKA-T structures revealed a conserved and identical substrate-binding site before and after maltose binding (Fig. 3b). Interestingly, despite the overall conservation of substrate binding at subsites −1 and −2 evidenced by identical coordination of the Asp-Glu-Asp catalytic triad residues (D213, E242, and D310) in all three TASKA structures (Fig. 3b), a slightly different configuration was found at the reducing ring of maltose in subsite −1 of TASKA-M and TASKA-T. While the electron density of the O1′ atom of maltose in TASKA-T is well ordered, weaker density was observed for TASKA-M. In addition, we also observed that the water molecule (W1) that hydrogen bonds to O1′ is identical for TASKA-Apo and TASKA-M but is shifted by 1.2 Å in TASKA-T (Fig. 3b). Another water molecule, W2, forms hydrogen bonds with O1 and O5 of the non-reducing ring of maltose. A similar water molecule was found in TASKA-Apo but not in TASKA-T (Fig. 3b). We initially expected identical structures for TASKA-M and TASKA-T since the final bound ligand is the same. Therefore, it is not known whether the slight changes in coordination and configuration for the disaccharide rings and water molecules at subsites −1 and −2 seen in these high resolution structures are caused by individual crystal conditions or are true indications of the post-substrate-hydrolysis stage (TASKA-T) or enzyme-product stage (TASKA-M).

In a separate analysis, we modelled the acarbose-derived pseudo-hexasaccharide into TASKA-T using structural superposition with GTA. The model shows that the inhibitor acarbose, which mimics a starch substrate, fits well into the TASKA catalytic site without any steric clashes at subsites −2, −1, or +1. The Asp-Glu-Asp catalytic triad is well positioned to interact with the sugar residues: D310 binds to and distorts the sugar ring at subsite −1, whereas D213 serves as a nucleophile by attacking the C1 atom on the distorted sugar, and E242 serves as an acid by donating a proton to the glycosidic bond between subsites −1 and +1 (Fig. 4). H217 is also strictly conserved to bind to subsite +1. On the other hand, subsite +2 in TASKA is not as well defined as in GTA. To bind the sugar residue at subsite +2 of TASKA, the aromatic side chain of the conserved W244 residue must first tilt ~60° to allow π-π stacking interactions with the sugar ring (Fig. 4). Second, the η4-α5 loop (F164–Q169) of domain B needs to move ~1.7 Å towards the subsite +2, presumably to stabilize substrate binding through the bulky side chain of W166 (Fig. 4). Furthermore, while K216, which replaces R220 in GTA, can still bind to the sugar residue at subsite +2, F178, which replaces Y182 in GTA, can no longer form a similar hydrogen bond to the sugar’s O6 atom. To accommodate the acarbose inhibitor, therefore, TASKA must adopt a conformational change upon ligand binding at subsite +2. Alternatively, subsite +2 of TASKA may have actually shifted towards the η4-α5 loop and adopted a different binding mode. In addition, shifting the η4-α5 loop widens the ligand-binding groove, and substitution of N171, L176, and R220 in GTA with the smaller A167, V172, and K216 residues, respectively, creates additional space, perhaps allowing for a subsite +3 not observed in GTA, which may also interact with the H245 residue that replaces S249 in GTA. Thus, in combination with the GTA-acarbose structure, the TASKA structures have provided insights into substrate binding at subsites +2 to −2 for this new subclass of glycosyl hydrolases.

**Figure 5:**
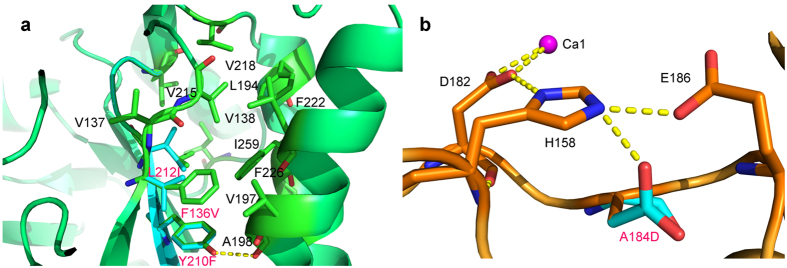
Model of TASKA mutants F136V, A184D, Y210F, and L212I. (**a**) The F136V and L212I mutants affect hydrophobic interactions in domain A, while Y210F may remove a hydrogen bond (yellow dotted line). (**b**) The A184D mutant may contribute a hydrogen bond between the carboxylate side chain and the side chains of H158 and E186 to interrupt the H158-E186 interaction.

A previous study examined the effects of mutagenesis in the CSR of full-length ASKA and characterized the thermostability following mutations at residues F113V^ASKA^, A161D^ASKA^, Y187F^ASKA^, and L189I^ASKA^ (ASKA numbering), equivalent to F136V, A184D, Y210F, and L212I herein^4^. F136V and A184D exhibited longer half-life thermostability, whereas Y210F and L212I had lower thermostability at 65 °C^7^. Our new X-ray structures of TASKA suggest explanations for the observed effects of mutagenesis. The TASKA structure shows that F136 and L212 are involved in hydrophobic interactions with residues V138, L194, Y210, V215, F222, W223, F226, L239, and I259, which are important for the structural stability of domain A (Fig. 5a). Mutation of the hydrophobic residues changes the hydrophobic interaction surface and affects the thermostability of the enzyme. The TASKA structure also indicates that the Y210F mutation may remove a hydrogen bond between Y210 and the main-chain oxygen atom of A198 (Fig. 5a), which explains the lower thermostability of the Y210F mutant. Modelling of A184D demonstrates that the carboxylate side chain is hydrogen bonded with the side chains of H158 and E186 (Fig. 5b), thereby disrupting the H158-E186 interaction. The A184D mutant showed higher catalytic activity and thermostability compare to the wild type. As H158 also binds to D182 (Fig. 5b), which in turn binds Ca1, this mutation may affect Ca1 binding. Ca1 contributes to structural stability, as does the A184D mutant (with a longer half-life at 65 °C); therefore, we hypothesize that A184D increases the Ca1 binding affinity by weakening the positive charge on H158 towards the D182 carboxylate, which results in a more stable structure between D182 and Ca1 and higher levels of enzyme activity.

In conclusion, the structures of TASKA-Apo and TASKA-maltose complexes complement that of the GTA-acarbose complex and provide insights into the conformational changes accompanying maltose binding at subsites −1 and −2 of GH13 α-amylases. Structural comparisons reveal conservation of substrate binding interactions at subsites −2, −1, and +1 but conformational changes at conserved residues W166 and W244 and loop η4-α5 of domain B to accommodate subsites +2, which might alternatively connect to an additional subsite +3 with a different ligand binding mode. The TASKA structure with four Ca^2+^ ion binding sites is unique among members of the GH13 family; however, the physiological relevance of Ca3 and Ca4 not yet confirmed. Our TASKA structures also suggest explanations for the effects of previously characterized ASKA mutants on enzyme thermostability. The structure-guided sequence alignment and phylogenetic analysis indicate a deviated α-amylase subgroup for *A. tepidamans*. To determine the physiological function of Ca3 and Ca4 of TASKA, examination of the residues involved in these binding sites via site-directed mutagenesis and biochemical analyses is needed. Finally, determination of the structure of an *A. tepidamans* α-amylase homologue thought to adopt another unique GH13 α-amylase structure would provide insight into the structural evolution of α-amylases.

## Methods

### Construction, expression, and purification of TASKA

A truncated α-amylase (EC 3.2.1.1) from *Anoxybacillus* sp. SK3-4 (TASKA) was constructed by removing 23 residues at the N-terminus (signal peptide, residues 1–23) and 27 residues at the C-terminus (residues 479–505) predicted to be a transmembrane region^9^ by TMHMM and SOSUI^36^,^37^. The TASKA gene was constructed by PCR with Q5 High Fidelity DNA polymerase (NEB, Ipswich, MA, USA) using primers with *Eco*RI and *Xho*I restriction sites (TASKA_F, 5′-CCTGAATTCAAAACGGAGC-3′ and TASKA_R, 5′-CCTCTCGAGGTTTAACCC-3′)^5^. The TASKA gene was cloned into pET-28a to produce the recombinant pET28a-TASKA plasmid with 6×-His-tags at the N- and C-termini. Recombinant TASKA was transformed into *E. coli* BL21 (DE3) cells, which were grown at 310 K with 200 rev/min agitation to OD_600_ ~ 0.5–0.6 prior to induction with 0.4 mM isopropyl-β-D-thiogalactopyranoside. The culture was incubated for additional 3 h before the cells were harvested and lysed using B-PER Protein Extraction Reagent (Thermo Fisher Scientific, Rockford, IL, USA). The cell lysate was dialyzed overnight against the purification binding buffer (0.5 M NaCl, 0.1 M imidazole, and 0.02 M sodium phosphate, pH 7.4) followed by protein purification using a 5-mL HisTrap column (GE Healthcare, Uppsala, Sweden). The protein was eluted using buffer containing 0.02 M sodium phosphate (pH 7.4), 0.5 M NaCl, and 0.5 M imidazole. The enzyme activity and purity of the collected fractions were determined by 3,5-dinitrosalicylic acid (DNS) assay and SDS-PAGE, respectively^8^.

### Crystallization and preliminary X-ray analysis of TASKA-Apo, TASKA-M, and TASKA-T

The pooled purified TASKA protein was buffer exchanged into 50 mM Tris (pH 8.0) using a Vivaspin concentrator fitted with a 5-kDa molecular weight cut-off filter (Sartorius Stedim Biotech, Göttingen, Germany) and concentrated to 10.8 mg/mL. Initial crystallization screening was carried out using the sitting drop vapour diffusion method in 96-well MRC Crystallization Plates^TM^ (Molecular Dimensions, Newmarket, UK) with crystal screen I and II kits (Hampton Research, Aliso Viejo, CA, USA). The drops containing 0.5 μL TASKA protein and 0.5 μL reservoir solution were equilibrated against 80 μL reservoir solution at 293 K. Crystal hits were identified for the reservoir condition containing 0.2 M calcium acetate, 0.1 M sodium cacodylate (pH 6.5), and 18% (*w/v*) polyethylene glycol 8,000. The condition was further optimized to obtain good diffraction-quality crystals. TASKA-Apo crystals that diffracted to 1.9 Å resolution were grown against reservoir containing 0.1 M calcium acetate, 0.1 M sodium cacodylate (pH 6.5), and 18% (*w/v*) polyethylene glycol 8,000. The crystals were flash-cooled with liquid nitrogen after soaking in cryoprotectant solution consisting of 0.1 M Tris-HCl (pH 8.0), 0.2 M calcium acetate, 0.1 M sodium cacodylate (pH 6.5), 19.8% (*w/v*) polyethylene glycol 8,000, and 16% (*v/v*) glycerol.

The TASKA-M and TASKA-T complexes were crystallized using the streak seeding method with a commercially available seeding tool (Hampton Research, Aliso Viejo, CA, USA). The seeding tool was dipped into a drop containing TASKA-Apo crystals and streaked on the drops that consisted of 0.3 μL 6 mg/mL TASKA protein and 0.3 μL reservoir solution (0.1 M calcium acetate, 0.1 M sodium cacodylate (pH 6.5), and 18% (*w/v*) polyethylene glycol 8,000 with 20 mM maltose or maltotriose). TASKA-T crystals were flash-cooled directly from the drop, while TASKA-M crystals were combined with 1 μL cryoprotectant solution (0.1 M Tris-HCl (pH 8.0), 0.2 M calcium acetate, 0.1 M sodium cacodylate (pH 6.5), 19.8% (*w/v*) polyethylene glycol 8,000, and 16% (*v/v*) glycerol) before flash cooling.

X-ray (wavelength 1.5418 Å) diffraction data were collected under a nitrogen gas stream at 100 K using an R-AXIS IV++ area detector on a Rigaku MicroMax-007 HF X-ray generator. All data sets were processed in space group P2_1_2_1_2_1_ with the XDS program package^38^. The TASKA-M structure was solved by molecular replacement with PHASER^39^ in CCP4 software suite^40^ using GTA (PDB ID: 4E2O) as a model, autobuilt with ArpWarp^41^, and refined and manually built using Refmac^42^ and Coot^43^. The TASKA-Apo and TASKA-T structures were then solved using TASKA-M as a model. The backbone dihedral angles of 99.8–100% of the residues of all three structures fall into the most favoured or allowed regions of the Ramachandran plot, as defined by PROCHECK^44^. Data collection and refinement statistics are listed in Table 1. Subsite numbering was in accordance with the nomenclature for enzymatic subsites of carbohydrate-processing enzymes proposed by Davies, *et al.*^45^.

### Endogeneous calcium determination by isothermal titration calorimetry

Purified TASKA was buffer exchanged against 50 mM HEPES-NaOH buffer (pH 8.0) and subsequently concentrated to 16.1 mg/mL using Amicon® Ultra Centrifugal filters with 10,000 Nominal Molecular Weight Limit (Merck, White House Station, NJ, USA). ITC experiments were carried out using a MicroCal PEAQ-ITC (Malvern Instruments, Malvern, United Kingdom) by transferring 0.28 mL concentrated TASKA into the sample cell. The concentrated TASKA was titrated with 1 mM CaCl_2_ at room temperature and subsequently titrated with 1 mM EDTA. The resulting thermogram was analysed using the manufacturer’s analysis software.

### Molecular dynamics simulation of TASKA-T

MD simulations of the TASKA-T structure with and without maltose bound were performed in GROMACS 4.5^46^. The topology and parameters of maltose were prepared in SwissParam^47^. The MD simulation was performed using a CHARMM27 force field. The systems were solvated with TIP3P water in a dodecahedral box with 10 Å between the protein structure and the box. The system was subsequently neutralised, energy minimised, and equilibrated in NVT and NPT ensembles. The temperature was maintained at 293 K. On completing system equilibration, a production run of 10 ns was performed.

## Additional Information

**How to cite this article**: Chai, K. P. *et al.* Crystal structure of *Anoxybacillus* a-amylase provides insights into maltose binding of a new glycosyl hydrolase subclass. *Sci. Rep.* **6**, 23126; doi: 10.1038/srep23126 (2016).

## Supplementary Material

Supplementary Information

## Figures and Tables

**Table 1:** Data collection and refinement statistics for TASKA-Apo, TASKA-M, and TASKA-T.

	TASKA-Apo	TASKA-M	TASKA-T
Space group	P2_1_2_1_2_1_	P2_1_2_1_2_1_	P2_1_2_1_2_1_
*a*, *b*, *c* (Å)	61.43, 63.44, 122.76	64.01, 65.30, 126.71	61.82, 63.53, 122.97
*α*, *β*, *γ* (°)	90, 90, 90	90, 90, 90	90, 90, 90
Resolution range (Å)	19.42–1.95 (2.00–1.95)*	19.60–1.85 (1.89–1.85)*	19.54–1.90 (1.94–1.90)*
*R*_*merge*_	0.065 (0.372)	0.063 (0.516)	0.089 (0.579)
*R*_*meas*_	0.079 (0.466)	0.073 (0.617)	0.104 (0.696)
*R*_p.i.m._	0.044 (0.275)	0.034 (0.328)	0.053 (0.377)
Total No. of unique reflections	34971	43446	37601
Completeness (%)	98.1 (93.4)	94.5 (83.6)	96.7 (86.6)
Redundancy	2.9 (2.5)	3.8 (2.8)	3.6 (2.9)
║*I*/*σ*(*I*)║	13.3/2.5	15.7/1.8	12.6/1.9
Refinement			
*R*_*work*_*/R*_*free*_	17.69/23.60	15.46/19.27	15.58/20.91
No. atoms			
Protein	3776	3787	3783
Ligand ion	–	23	23
12	4	4	
Water	405	412	518
B-factors			
Protein	19.9	18.1	16.5
Ligand ion	–	36.5	28.2
29.9	17.1	18.8	
Water	37.4	27.2	31.8
RMS deviations			
Bond angles (°)	2.2318	1.8962	1.8206
Bond length (Å)	0.0226	0.0190	0.0182

^*^The parentheses indicate the values for the highest resolution shell.
