# Supplementary material for: Crystal structure of Anoxybacillus α-amylase provides insights into maltose binding of a new glycosyl hydrolase subclass
Source: Sci Rep. 2016 Mar 15;6:23126. doi: 10.1038/srep23126 (PMC4791539; doi:10.1038/srep23126)
Supplement: Supplementary Information [file srep23126-s1.pdf]

Supplementary Information

Crystal structure of *Anoxybacillus* α-amylase provides insights into maltose binding of a new glycosyl hydrolase subclass

Kian Piaw Chai[1+], Noor Farhan Binti Othman[2], Aik-Hong Teh[3], Kok Lian Ho[4], Kok-Gan Chan[5], Mohd Shahir Shamsir[1], Kian Mau Goh[1+*], Chyan Leong Ng[2,+*]

[1] Universiti Teknologi Malaysia, Faculty of Biosciences and Medical Engineering, 81310 Skudai, Johor, Malaysia.

[2] Universiti Kebangsaan Malaysia, Institute of Systems Biology, 43600 UKM Bangi, Selangor, Malaysia.

[3] Universiti Sains Malaysia, Centre for Chemical Biology, 11800 Penang, Malaysia.

[4] Universiti Putra Malaysia, Department of Pathology, Faculty of Medicine and Health Sciences, 43400 Serdang, Selangor, Malaysia.

[5] University of Malaya, Division of Genetics and Molecular Biology, Institute of Biological Sciences, Faculty of Science, 50603 Kuala Lumpur, Malaysia.

* Corresponding author: clng@ukm.edu.my, gohkianmau@utm.my

Table S1 Properties and structures of TASKA and related GHs.

Species	Enzyme	GH	PDB	Resolution (Å)	Seq. identity (%)*	RMSD (Å)*	Total domain	Number of Ca bound	Function of Ca	Enzyme opt. temp. (°C)/ pH	Product specificity	Reference
Anoxybacillus sp. SK3-4	α-amylase	GH13	5A2A	1.9	100	0	3	4	Activity & stability	60/ 8.0	Maltooligosaccharides (mainly G2)	[1,2]
G. thermoleovorans	α-amylase	GH13	4E2O	2.1	73	1.1	3	2	Stability	70/ 6.0	Maltooligosaccharides (mainly G2)	[3]
Aspergillus niger	α-amylase	GH13_5	2GVY	1.8	32	1.9	3	1	Stability	60/ 4.0	Maltooligosaccharides	[4-6]
G. sterarothermophilus	Maltogenic α-amylase	GH13_2	1QHO	1.7	32	2.0	5	3	Activity & stability	60/ 6.0	Maltooligosaccharides (mainly G2)	[6-8]
G. stearothermophilus	Neopulullanase	GH13_20	1J0H	1.9	34	2.0	4	1	n.d	60/ 6.0	Panose, maltose, glucose	[9,10]
B. circulans	CGTase	GH13_2	1EO5	2.0	29	2.1	5	3	Activity & stability	50/ 6.0	Cyclodextrin (mainly β-CD)	[11-13]
Hordeum vulgare	α-amylase	GH13_6	1RPK	2.0	18	2.5	3	3	Activity & stability	37/ 5.5	Maltooligosaccharides	[14,15]
Halothermothrix orenii	α-amylase	GH13	3BCF	2.3	22	2.9	4	4	As confactor	65/ 8.0	Maltooligosaccharides	[16]

B. amyloliquefaciens	α-amylase	GH13_5	3BH4	1.4	32	3.0	3	4	Activity & stability	55/ 6.0	Maltooligosaccharides	[17,18]

References

1 Ranjani, V. *et al.* Protein engineering of selected residues from conserved sequence regions of a novel *Anoxybacillus* α-amylase. *Sci. Rep.* **4**, doi:10.1038/srep05850 (2014).

2 Chai, Y. Y., Rahman, R. N. Z. R. A., Illias, R. M. & Goh, K. M. Cloning and characterization of two new thermostable and alkalitolerant α-amylases from the *Anoxybacillus* species that produce high levels of maltose. *J. Ind. Microbiol. Biotechnol.* **39**, 731–741, doi:10.1007/s10295-011-1074-9 (2012).

3 Mok, S.-C., Teh, A.-H., Saito, J. A., Najimudin, N. & Alam, M. Crystal structure of a compact α-amylase from *Geobacillus thermoleovorans*. *Enzyme Microb. Technol.* **53**, 46–54, doi:10.1016/j.enzmictec.2013.03.009 (2013).

4 Vujičić-Žagar, A. & Dijkstra, B. W. Monoclinic crystal form of *Aspergillus niger* α-amylase in complex with maltose at 1.8 Å resolution. *Acta Crystallogr. Sect. F Struct. Biol. Cryst. Commun.* **62**, 716–721, doi:10.1107/S1744309106024729 (2006).

5 Sahnoun, M. *et al.* Production, purification and characterization of two α-amylase isoforms from a newly isolated *Aspergillus oryzae* strain S2. *Process Biochem.* **47**, 18–25, doi:10.1016/j.procbio.2011.09.016 (2012).

6 Kindle, K. Characteristics and production of thermostable α-amylase. *Appl. Biochem. Biotechnol.* **8**, 153–170, doi:10.1007/bf02778096 (1983).

7 Dauter, Z. *et al.* X-ray structure of Novamyl, the five-domain “maltogenic” α-amylase from *Bacillus stearothermophilus*: maltose and acarbose complexes at 1.7 Å Resolution. *Biochemistry* **38**, 8385–8392, doi:10.1021/bi990256l (1999).

8 Al-Qodah, Z. Production and characterization of thermostable α-amylase by thermophilic *Geobacillus stearothermophilus*. *Biotechnol. J.* **1**, 850–857, doi:10.1002/biot.200600033 (2006).

9 Hondoh, H., Kuriki, T. & Matsuura, Y. Three-dimensional structure and substrate binding of *Bacillus stearothermophilus* neopullulanase. *J. Mol. Biol.* **326**, 177–188, doi:10.1016/S0022-2836(02)01402-X (2003).

10 Kuriki, T., Okada, S. & Imanaka, T. New type of pullulanase from *Bacillus stearothermophilus* and molecular cloning and expression of the gene in *Bacillus subtilis*. *J. Bacteriol.* **170**, 1554–1559 (1988).

11 Lawson, C. L. *et al.* Nucleotide sequence and X-ray structure of cyclodextrin glycosyltransferase from *Bacillus circulans* strain 251 in a maltose-dependent crystal Form. *J. Mol. Biol.* **236**, 590–600, doi:10.1006/jmbi.1994.1168 (1994).

12 Uitdehaag, J. C. M., van Alebeek, G.-J. W. M., van der Veen, B. A., Dijkhuizen, L. & Dijkstra, B. W. Structures of maltohexaose and maltoheptaose bound at the donor sites of cyclodextrin glycosyltransferase give insight into the mechanisms of transglycosylation activity and cyclodextrin size specificity. *Biochemistry* **39**, 7772–7780, doi:10.1021/bi000340x (2000).

13 Vassileva, A., Atanasova, N., Ivanova, V., Dhulster, P. & Tonkova, A. Characterization of cyclodextrin glucanotransferase from *Bacillus circulans* ATCC 21783 in terms of cyclodextrin production. *Ann. Microbiol.* **57**, 609–615, doi:10.1007/bf03175362 (2007).

14 Robert, X., Haser, R., Mori, H., Svensson, B. & Aghajari, N. Oligosaccharide binding to barley α-amylase 1. *J. Biol. Chem.* **280**, 32968–32978, doi:10.1074/jbc.M505515200 (2005).

15 Yuk, J.-B. *et al.* Effects of calcium ion concentration on starch hydrolysis of barley α-amylase isozymes. *J. Microbiol. Biotechnol.* **18**, 730–734 (2008).
16 Tan, T.-C., Mijts, B. N., Swaminathan, K., Patel, B. K. C. & Divne, C. Crystal structure of the polyextremophilic α-amylase AmyB from *Halothermothrix orenii*: Details of a productive enzyme–substrate complex and an N domain with a role in binding raw starch. *J. Mol. Biol.* **378**, 852–870, doi:10.1016/j.jmb.2008.02.041 (2008).
17 Alikhajeh, J. *et al.* Structure of *Bacillus amyloliquefaciens* α-amylase at high resolution: implications for thermal stability. *Acta Crystallogr. Sect. F Struct. Biol. Cryst. Commun.* **66**, 121–129, doi:10.1107/S1744309109051938 (2010).
18 Gangadharan, D., Nampoothiri, K. M., Sivaramakrishnan, S. & Pandey, A. Biochemical characterization of raw-starch-digesting α-amylase purified from *Bacillus amyloliquefaciens*. *Appl. Biochem. Biotechnol.* **158**, 653–662, doi:10.1007/s12010-008-8347-4 (2009).

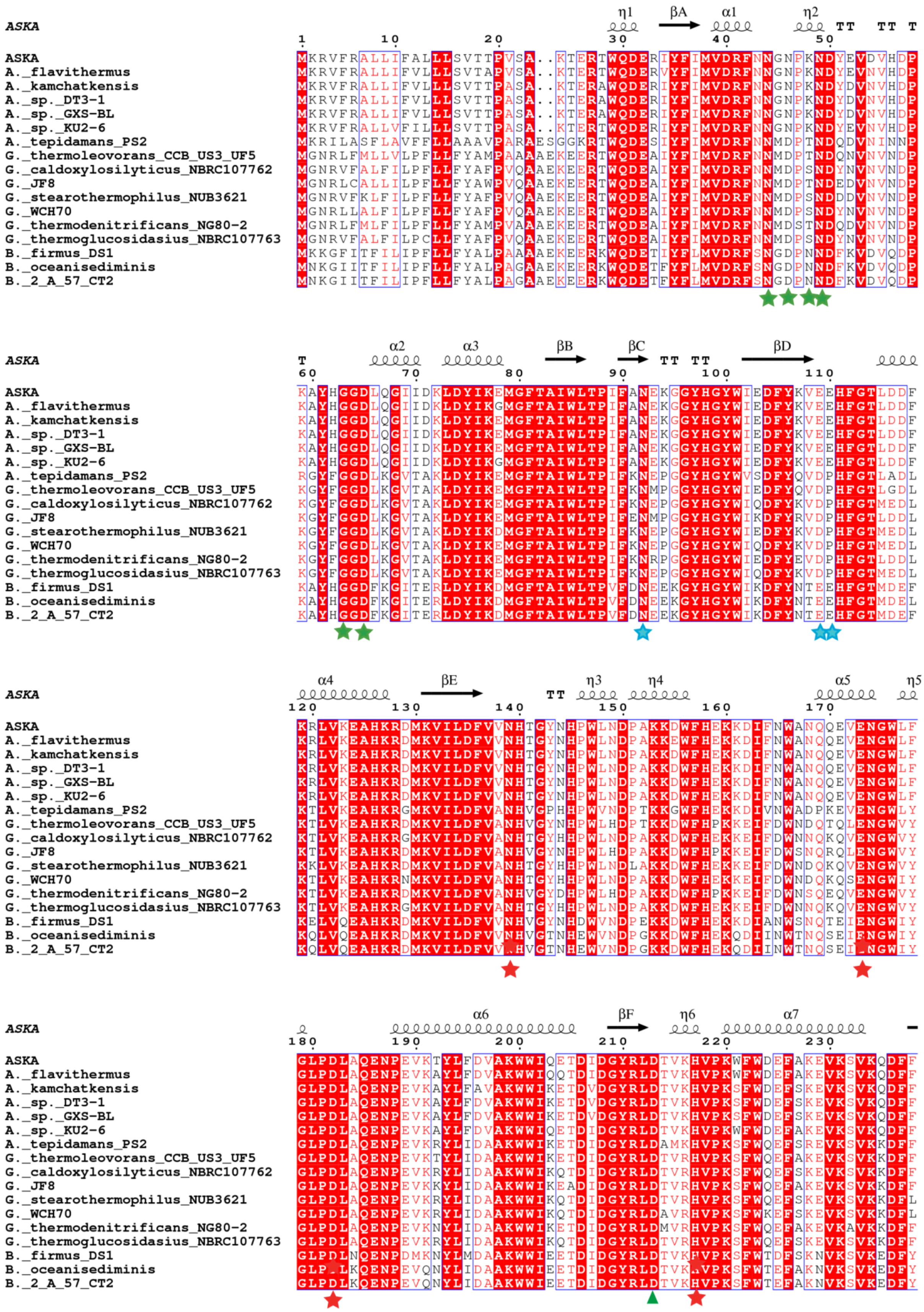

ASKA
η1 βA α1 η2 TT TT T
1 10 20 30 40 50
ASKA MKRVFRALLIFALLLSVTTPVSA..KTERTWQDERIYFIMVDRFNNGNPKNDYEVDVHDP
A._flavithermus MKRVFRALLIFVLLLSVTAPVSA..KTERTWQDERVYFIMVDRFNNGNPKNDYEVNVHDP
A._kamchatkensis MKRVFRALLIFVLLLSVTTPASA..KTERAWQDERIYFIMVDRFNNGNPKNDYDVNVHDP
A._sp._DT3-1 MKRVFRALLIFVLLLSVTTPASA..KTERAWQDERIYFIMVDRFNNGNPKNDYDVNVHDP
A._sp._GXS-BL MKRVFRALLIFVLLLSVTTPASA..KTERAWQDERIYFIMVDRFNNGNPKNDYDVNVHDP
A._sp._KU2-6 MKRVFRALLVFILLLSVTTPASA..KTERTWQDERIYFIMVDRFNNGNPKNDYEVNVHDP
A._tepidamans_PS2 MKRILASFLAVFFLLAAAVPARAESGGKRTWQDESIYFIMVDRFNNMDPTNDQDVNINNP
G._thermoleovorans_CCB_US3_UF5 MGNRLFMLLVLPFLLFYAMPAAAAEKEERTWQDEAIYFIMVDRFNNMDPTNDQNVNVNDP
G._caldoxylosilyticus_NBRC107762 MGNRVFALFILPFLLFYAFPVQAAEKEERTWQDEAIYFIMVDRFNNMDPSNDYNVNVNDP
G._JF8 MGNRLCALLILPFLLFYAWPVQAAEKEERTWQDEAIYFIMVDRFNNMDPTNDEDVNVNDP
G._stearothermophilus_NUB3621 MGNRVFKLFILPFLLFYAFPVQAAEKEERTWQDEAIYFIMVDRFNNMDPSNDDDVNVNDP
G._WCH70 MGNRLLALFILPFLLFYAFPVQAAEKEERTWQDEAIYFIMVDRFNNMDPSNDYNVNVNDP
G._thermodenitrificans_NG80-2 MGNRLFMLFILPFLLFYAMPVAAAEKEERTWQDEAIYFIMVDRFNNMDSTNDQDVNVNDP
G._thermoglucosidasius_NBRC107763 MGNRVFALFILPCLLFYAFPVQAAEKEERTWQDEAIYFIMVDRFNNMDPSNDYNVNVNDP
B._firmus_DS1 MKKGFITFILIPFLLFYALPAAAAEKEERKWQDETIYFLMVDRFSNGDPNNDFKVDVQDP
B._oceanisediminis MNKGIITFILIPFLLFYALPAGAAEKEERKWQDETFYFLMVDRFSNGDPNNDFKVDVQDP
B._2_A_57_CT2 MNKGIITFILIPFLLFYALPAGAAEKEERKWQDETFYFLMVDRFSNGDPNNDFKVDVQDP
ASKA
T α2 α3 βB βC TT TT βD
60 70 80 90 100 110
ASKA KAYHGGDLQGIIDKLDYIKEMGFTAIWLTPIFANEKGGYHGYWIEDFYKVEEHFGTLDDF
A._flavithermus KAYHGGDLQGIIDKLDYIKGMGFTAIWLTPIFANEKGGYHGYWIEDFYKVEEHFGTLDDF
A._kamchatkensis KAYHGGDLQGIIDKLDYIKEMGFTAIWLTPIFANEKGGYHGYWIEDFYKVEEHFGTLDDF
A._sp._DT3-1 KAYHGGDLQGIIDKLDYIKEMGFTAIWLTPIFANEKGGYHGYWIEDFYKVEEHFGTLDDF
A._sp._GXS-BL KAYHGGDLQGIIDKLDYIKEMGFTAIWLTPIFANEKGGYHGYWIEDFYKVEEHFGTLDDF
A._sp._KU2-6 KAYHGGDLQGIIDKLDYIKGMGFTAIWLTPIFANEKGGYHGYWIEDFYKVEEHFGTLDDF
A._tepidamans_PS2 RGYFGGDLKGVTAKLDYIKEMGFTAIWLTPIFKNEPGGYHGYWVSDFYQVDPHFGTLADL
G._thermoleovorans_CCB_US3_UF5 KGYFGGDLKGVTAKLDYIKEMGFTAIWLTPIFKNMPGGYHGYWIEDFYQVDPHFGTLGDL
G._caldoxylosilyticus_NBRC107762 KGYFGGDLKGVTAKLDYIKEMGFTAIWLTPIFKNEPGGYHGYWIQDFYKVDPHFGTMEDL
G._JF8 KGYFGGDLKGVTAKLDYIKEMGFTAIWLTPIFENMPGGYHGYWIKDFYRVDPHFGTLDDL
G._stearothermophilus_NUB3621 KGYFGGDLKGVTAKLDYIKDMGFTAIWLTPIFKNEPGGYHGYWIEDFYKVDPHFGTMEDL
G._WCH70 KGYFGGDLKGVTAKLDYIKEMGFTAIWLTPIFKNEPGGYHGYWIQDFYKVDPHFGTMEDL
G._thermodenitrificans_NG80-2 KGYFGGDLKGVTAKLDYIKEMGFTAIWLTPIFKNRPGGYHGYWIEDFYEVDPHFGTLDDL
G._thermoglucosidasius_NBRC107763 KGYFGGDLKGVTAKLDYIKEMGFTAIWLTPIFKNEPGGYHGYWIQDFYKVDPHFGTMEDL
B._firmus_DS1 KAYHGGDFKGITERLDYIKDMGFTAIWLTPVFDNEEKGYHGYWIKDFYNTEEHFGTMDEF
B._oceanisediminis KAYHGGDFKGITERLDYIKDMGFTAIWLTPVFDNEEKGYHGYWIKDFYNTEEHFGTMDEL
B._2_A_57_CT2 KAYHGGDFKGITERLDYIKDMGFTAIWLTPVFDNEEKGYHGYWIKDFYNTEEHFGTMDEF
ASKA
α4 βE TT η3 η4 α5 η5
120 130 140 150 160 170
ASKA KRLVKEAHKRDMKVILDFVVNHTGYNHPWLNDPAKKDWFHEKKDIFNWANQQEVENGWLF
A._flavithermus KRLVKEAHKRDMKVILDFVVNHTGYNHPWLNDPAKKEWFHEKKDIFNWANQQEVENGWLF
A._kamchatkensis KRLVKEAHKRDMKVILDFVVNHTGYNHPWLNDPAKKDWFHEKKDIFNWANQQEVENGWLF
A._sp._DT3-1 KRLVKEAHKRDMKVILDFVVNHTGYNHPWLNDPAKKDWFHEKKDIFNWANQQEVENGWLF
A._sp._GXS-BL KRLVKEAHKRDMKVILDFVVNHTGYNHPWLNDPAKKDWFHEKKDIFNWANQQEVENGWLF
A._sp._KU2-6 KRLVKEAHKRDMKVILDFVVNHTGYNHPWLNDPAKKDWFHEKKDIFNWANQQEVENGWLF
A._tepidamans_PS2 KTLVKEAHKRGMKVILDFVANHVGPHHPWVNDPTKKGWFHEKKDIVNWADPKEVENGWLY
G._thermoleovorans_CCB_US3_UF5 KTLVKEAHKRDMKVILDFVANHVGYNHPWLHDPTKKDWFHPKKEIFDWNDQTQLENGWVY
G._caldoxylosilyticus_NBRC107762 KTLVKEAHKRGMKVILDFVANHTGYHHPWLNDPAKKDWFHEKKEIFDWNNQKQVENGWVY
G._JF8 KTLVKEAHKRDMKVILDFVANHVGYNHPWLHDPAKKDWFHPKKEIFDWSNQRQLENGWVY
G._stearothermophilus_NUB3621 KKLVKEAHKRDMKVILDFVANHTGYHHPWLNDLAKKDWFHEKKEIFDWNDQKQVENGWVY
G._WCH70 KTLVKEAHKRNMKVILDFVANHTGYHHPWLNDPAKKDWFHEKKEIFDWNDQKQSENGWIY
G._thermodenitrificans_NG80-2 KTLVKEAHKRDMKVILDFVANHVGYDHPWLHDPAKKDWFHPKKEIFDWNSQEQVENGWVY
G._thermoglucosidasius_NBRC107763 KTLVKEAHKRGMKVILDFVANHTGYHHPWLNDPAKKDWFHEKKEIFDWNNQKQVENGWVY
B._firmus_DS1 KELVQEAHKRDMKVILDFVVNHVGYNHDWVNDPEKKDWFHEKKDIANWSNQTEIENGWIY
B._oceanisediminis KQLVQEAHKRDMKVILDFVVNHVGTNHEWVNDPGKKDWFHEKQDIINWTNQSEIENGWIY
B._2_A_57_CT2 KQLVQEAHKRDMKVILDFVV[illegible]HVGTNHEWVNDPGKKDWFHEKQDIINWTNQSEI[illegible]NGWIY
ASKA
α6 βF η6 α7
180 190 200 210 220 230
ASKA GLPDLAQENPEVKTYLFDVAKWWIQETDIDGYRLDTVKHVPKWFWDEFAKEVKSVKQDFF
A._flavithermus GLPDLAQENPEVKAYLFDVAKWWIQQTDIDGYRLDTVKHVPKWFWDEFAKEVKSVKQDFF
A._kamchatkensis GLPDLAQENPEVKAYLFAVAKWWIKETDVDGYRLDTVKHVPKSFWDEFSKEVKSVKQDFF
A._sp._DT3-1 GLPDLAQENPEVKAYLFDVAKWWIKETDVDGYRLDTVKHVPKSFWDEFSKEVKSVKQDFF
A._sp._GXS-BL GLPDLAQENPEVKAYLFDVAKWWIKETDVDGYRLDTVKHVPKSFWDEFSKEVKSVKQDFF
A._sp._KU2-6 GLPDLAQENPEVKAYLFDVAKWWIQETDIDGYRLDTVKHVPKWFWDEFAKEVKSVKQDFF
A._tepidamans_PS2 GLPDLAQENPEVKRYLIDAAKWWIKETDIDGYRLDAMKHVPKSFWQEFSREVKSVKKDFF
G._thermoleovorans_CCB_US3_UF5 GLPDLAQENPEVKTYLIDAAKWWIKETDIDGYRLDTVRHVPKSFWQEFAKEVKSVKKDFF
G._caldoxylosilyticus_NBRC107762 GLPDLAQENPEVKRYLIDAAKWWIKQTDIDGYRLDTVRHVPKSFWQEFSKEVKSVKKDFF
G._JF8 GLPDLAQENPEVKKYLIDAAKWWIKEADIDGYRLDTVRHVPKSFWQEFAKEVKSVKKDFF
G._stearothermophilus_NUB3621 GLPDLAQENPEVKRYLIDAAKWWIKQTDIDGYRLDTVRHVPKSFWQEFSKEVKSVKKDFL
G._WCH70 GLPDLAQENPEVKRYLIDAAKWWIKQTDIDGYRLDAVRHVPKSFWKEFSKEVKSVKKDFL
G._thermodenitrificans_NG80-2 GLPDLAQENPEVKNYLIDAAKWWIKETDIDGYRLDMVRHVPKSFWQEFAKEVKAVKKDFF
G._thermoglucosidasius_NBRC107763 GLPDLAQENPEVKRYLIDAAKWWIKQTDIDGYRLDTVRHVPKSFWQEFSKEVKSVKKDFF
B._firmus_DS1 GLPDLNQENPDMKNYLMDAAKWWIEETDIDGYRLDTVKHVPKSFWTDFSKNVKSVKEDFY
B._oceanisediminis GLP[illegible]LKQENPEVQNYLIDAAKWWIEETDIDGYRLDTVK[illegible]VPKSFWTEFAKNVKSVKEDFY
B._2_A_57_CT2 GLPDLKQENPEVQNYLIDAAKWWIEETDIDGYRLDTVKHVPKSFWTEFAKNVKSVKEDFY

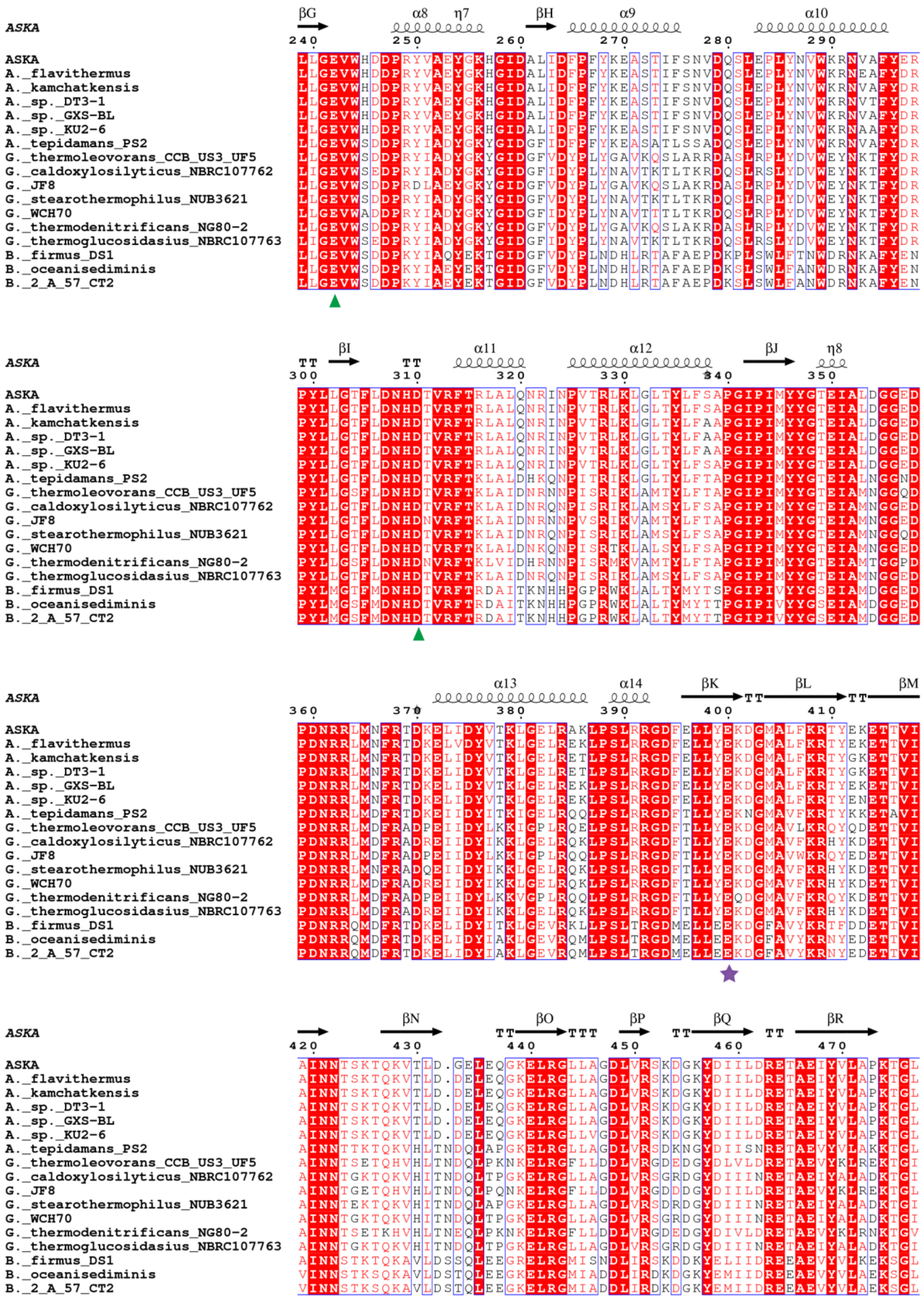
ASKA
βG α8 η7 βH α9 α10
240 250 260 270 280 290
ASKA LLGEVWHDDPRYVAEYGKHGIDALIDFPFYKEASTIFSNVDQSLEPLYNVWKRNVAFYER
A._flavithermus LLGEVWHDDPRYVAEYGKHGIDALIDFPFYKEASTIFSNVDQSLEPLYNVWKRNEAFYDR
A._kamchatkensis LLGEVWHDDPRYVAEYGKHGIDALIDFPFYKEASTIFSNVDQSLEPLYNVWKRNVAFYDR
A._sp._DT3-1 LLGEVWHDDPRYVAEYGKHGIDALIDFPFYKEASTIFSNVDQSLEPLYNVWKRNVTFYDR
A._sp._GXS-BL LLGEVWHDDPRYVAEYGKHGIDALIDFPFYKEASTIFSNVDQSLEPLYNVWKRNVAFYDR
A._sp._KU2-6 LLGEVWHDDPRYVAEYGKHGIDALIDFPFYKEASTIFSNVDQSLEPLYNVWKRNAAFYDR
A._tepidamans_PS2 LLGEVWSDDPRYIADYGKYGIDGFIDFPFYKEASATLSSADQSLEPLYNVWKRNVAFYDR
G._thermoleovorans_CCB_US3_UF5 LLGEVWSDDPRYIADYGKYGIDGFVDYPLYGAVKQSLARRDASLRPLYDVWEYNKTFYDR
G._caldoxylosilyticus_NBRC107762 LIGEVWSEDPRYIADYGKYGIDGFIDYPLYNAVTKTLTKRDQSLRSLYDVWEYNKTFYDR
G._JF8 LLGEVWSDDPRDLAEYGKYGIDGFVDYPLYGAVKQSLAKRDASLRPLYDVWEYNKTFYDR
G._stearothermophilus_NUB3621 LLGEVWSDDPRYIADYGKYGIDGFVDYPLYNAVTKTLTKRDQSLRPLYDVWEYNKTFYDR
G._WCH70 LLGEVWADDPRYIADYGKYGIDGFIDYPLYNAVTTTLTKRDQSLRPLYDVWEYNKTFYDR
G._thermodenitrificans_NG80-2 LLGEVWSDDPRYIADYGKYGIDGFVDYPLYGAVKQSLAKRDASLRPLYDVWEYNKTFYDR
G._thermoglucosidasius_NBRC107763 LIGEVWSEDPRYIADYGKYGIDGFIDYPLYNAVTKTLTKRDQSLRSLYDVWEYNKTFYDR
B._firmus_DS1 LLGEVWSDDPKYIAQYEKTGIDGFVDYPLNDHLRTAFAEPDKPLSWLFTNWDRNKAFYEN
B._oceanisediminis LLGEVWSDDPKYIAEYEKTGIDGFVDYPLNDHLRTAFAEPDKSLSWLFANWDRNKAFYEN
B._2_A_57_CT2 LLGEVWSDDPKYIAEYEKTGIDGFVDYPLNDHLRTAFAEPDKSLSWLFANWDRNKAFYEN
ASKA
TT βI TT α11 α12 βJ η8
300 310 320 330 340 350
ASKA PYLLGTFLDNHDTVRFTRLALQNRINPVTRLKLGLTYLFSAPGIPIMYYGTEIALDGGED
A._flavithermus PYLLGTFLDNHDTVRFTRLALQNRINPVTRLKLGLTYLFSAPGIPIMYYGTEIALDGGED
A._kamchatkensis PYLLGTFLDNHDTVRFTRLALQNRINPVTRLKLGLTYLFAAPGIPIMYYGTEIALDGGED
A._sp._DT3-1 PYLLGTFLDNHDTVRFTRLALQNRINPVTRLKLGLTYLFAAPGIPIMYYGTEIALDGGED
A._sp._GXS-BL PYLLGTFLDNHDTVRFTRLALQNRINPVTRLKLGLTYLFAAPGIPIMYYGTEIALDGGED
A._sp._KU2-6 PYLLGTFLDNHDTVRFTRLALQNRINPVTRLKLGLTYLFSAPGIPIMYYGTEIALDGGED
A._tepidamans_PS2 PYLLGTFLDNHDTVRFTKLALDHKQNPITRIKLGLTYLFTAPGIPIMYYGTEIALNGGND
G._thermoleovorans_CCB_US3_UF5 PYLLGSFLDNHDTVRFTKLAIDNRNNPISRIKLAMTYLFTAPGIPIMYYGTEIAMNGGQD
G._caldoxylosilyticus_NBRC107762 PYLLGTFLDNHDTVRFTKLAIDNRQNPISRIKLAMSYLFSAPGIPIMYYGTEIAMNGGED
G._JF8 PYLLGTFLDNHDNVRFTKLAIDNRNNPVSRIKVAMTYLFTAPGIPIMYYGTEIAMNGGED
G._stearothermophilus_NUB3621 PYLLGTFLDNHDTVRFTKLAIDNRQNPISRIKLAMSYLFSAPGIPIMYYGTEIAMNGGQD
G._WCH70 PYLLGTFLDNHDTVRFTKLALDNKQNPISRTKLALSYLFSAPGIPIMYYGTEIAMNGGED
G._thermodenitrificans_NG80-2 PYLLGSFLDNHDNVRFTKLVIDHRNNPISRMKVAMTYLFTAPGIPIMYYGTEIAMTGGPD
G._thermoglucosidasius_NBRC107763 PYLLGTFLDNHDTVRFTKLAIDNRQNPISRIKLAMSYLFSAPGIPIMYYGTEIAMNGGED
B._firmus_DS1 PYLMGTFMDNHDTVRFTRDAITKNHHPGPRWKLALTYMYTSPGIPIVYYGSEIAMDGGED
B._oceanisediminis PYLMGSFMDNHDTVRFTRDAITKNHHPGPRWKLALTYMYTTPGIPIVYYGSEIAMDGGED
B._2_A_57_CT2 PYLMGSFMDNHDTVRFTRDAITKNHHPGPRWKLALTYMYTTPGIPIVYYGSEIAMDGGED
ASKA
α13 α14 βK TT βL TT βM
360 370 380 390 400 410
ASKA PDNRRLMNFRTDKELIDYVTKLGELRAKLPSLRRGDFELLYEKDGMALFKRTYEKETTVI
A._flavithermus PDNRRLMNFRTDKELVDYVTKLGELREKLPSLRRGDFELLYEKDGMALFKRTYEKETTVI
A._kamchatkensis PDNRRLMNFRTDKELIDYVTKLGELRETLPSLRRGDFELLYEKDGMALFKRTYGKETTVI
A._sp._DT3-1 PDNRRLMNFRTDKELIDYVTKLGELRETLPSLRRGDFELLYEKDGMALFKRTYGKETTVI
A._sp._GXS-BL PDNRRLMNFRTDKELIDYVTKLGELREKLPSLRRGDFELLYEKDGMALFKRTYEKETTVI
A._sp._KU2-6 PDNRRLMNFRTDKELIDYVTKLGELREKLPSLRRGDFELLYEKDGMALFKRTYENETTVI
A._tepidamans_PS2 PDNRRLMDFRTDKEIIDYITKLGELRQQLPSLRRGDFTLLYEKNGMAVFKRTYKKETAVI
G._thermoleovorans_CCB_US3_UF5 PDNRRLMDFRADPEIIDYLKKIGPLRQELPSLRRGDFTLLYEKDGMAVLKRQYQDETTVI
G._caldoxylosilyticus_NBRC107762 PDNRRLMDFRADREIIDYIKKLGELRQKLPSLRRGDFTLLYEKDGMAVFKRHYKDETTVI
G._JF8 PDNRRLMDFRADPEIIDYLKKIGPLRQQLPSLRRGDFTLLYEKDGMAVWKRQYEDETTVI
G._stearothermophilus_NUB3621 PDNRRLMNFRADQEIIDYIKKLGELRQKLPSLRRGDFTLLYEKDGMAVFKRHYKDETTVI
G._WCH70 PDNRRLMDFRADREIIDYIKKLGELRQKLPSLRRGDFTLLYEKDGMAVFKRHYKDETTVI
G._thermodenitrificans_NG80-2 PDNRRLMDFRADPEIIDYLKKVGPLRQQLPSLRRGDFTLLYEQDGMAVFKRQYKDETTVI
G._thermoglucosidasius_NBRC107763 PDNRRLMDFRADREIIDYIKKLGELRQKLPSLRRGDFTLLYEKDGMAVFKRHYKDETTVI
B._firmus_DS1 PDNRRQMDFRTDKELIDYITKLGEVRKLLPSLTRGDMELLEEKDGFAVYKRTFDDETTVI
B._oceanisediminis PDNRRQMDFRTDKELIDYIAKLGEVRQMLPSLTRGDMELLEEKDGFAVYKRNYEDETTVI
B._2_A_57_CT2 PDNRRQMDFRTDKELIDYIAKLGEVRQMLPSLTRGDMELLEEKDGFAVYKRNYEDETTVI
ASKA
βN TT βO TTT βP TT βQ TT βR
420 430 440 450 460 470
ASKA AINNTSKTQKVTLD.GELEQGKELRGLLAGDLVRSKDGKYDIILDRETAEIYVLAPKTGL
A._flavithermus AINNTSKTQKVTLD.DELEQGKELRGLLAGDLVRSKDGKYDIILDRETAEIYVLAPKTGL
A._kamchatkensis AINNTSKTQKVTLD.DELEQGKELRGLLAGDLVRSKDGKYDIILDRETAEIYVLAPKTGL
A._sp._DT3-1 AINNTSKTQKVTLD.DELEQGKELRGLLAGDLVRSKDGKYDIILDRETAEIYVLAPKTGL
A._sp._GXS-BL AINNTSKTQKVTLD.DELEQGKELRGLLAGDLVRSKDGKYDIILDRETAEIYVLAPKTGL
A._sp._KU2-6 AINNTSKTQKVTLD.DELEQGKELRGLLVGDLVRSKDGKYDIILDRETAEIYVLAPKTGL
A._tepidamans_PS2 AINNTTKTQKVHLTNDQLAPGKELRGLLAGDLVRSDKNGYDIISNRETAEVYVLAKKTGL
G._thermoleovorans_CCB_US3_UF5 AINNTSETQHVHLTNDQLPKNKELRGFLLDDLVRGDEDGYDLVLDRETAEVYKLREKTGI
G._caldoxylosilyticus_NBRC107762 AINNTGKTQKVHITNDQLTPGKELRGLLAGDLVRSGRDGYDIIINRETAEIYALADKTGI
G._JF8 AINNTGETQHVHLTNDQLPQNKELRGFLLDDLVRGDDDGYDIILDRETAEVYKLREKTGI
G._stearothermophilus_NUB3621 AINNTEKTQKVHITNDQLAPGKELRGLLAGDLVRSDRDGYDIIINRETAEIYALADKTGI
G._WCH70 AINNTGKTQKVHITNDQLTPGKELRGLLAGDLVRSGRDGYDIIINRETAEIYALADKTGI
G._thermodenitrificans_NG80-2 AINNTSETKHVHLTNEQLPKNKELRGFLLDDLVRGDEDGYDIVLDRETAEVYKLRNKTGV
G._thermoglucosidasius_NBRC107763 AINNTGKTQKVHITNDQLTPGKELRGLLAGDLVRSGRDGYDIIINRETAEIYALADKTGI
B._firmus_DS1 AINNSTKTQKAVLDSSQLEEGRELRGMISNDLIRSKDDKYELIIDREEAEVYVLKEKSGL
B._oceanisediminis VINNSTKSQKAVLDSTQLEEGKELRGMIADDLIRDKDGKYEMIIDREEAEVYVLAEKSGL
B._2_A_57_CT2 VINNSTKSQKAVLDSTQLEEGKELRGMIADDLIRDKDGKYEMIIDREEAEVYVLAEKSGL

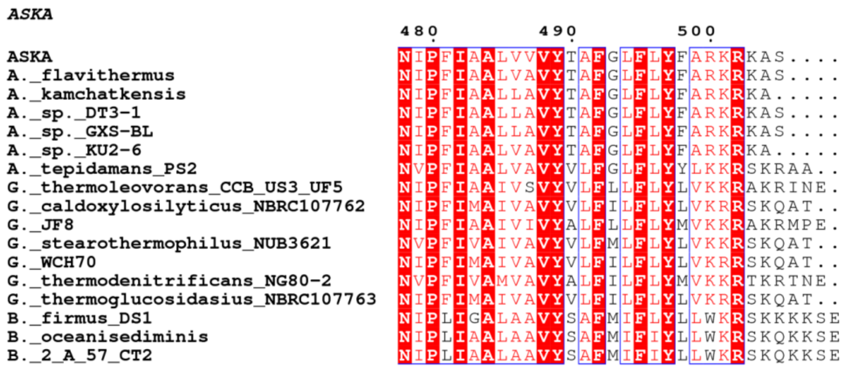

Fig. S1 Structure-based sequence alignment of ASKA-amylase and its homologs. The homologs are classified to *Anoxybacillus* species (*A. flavithermus*, *A. kamchatkensis*, *A.* sp. DT3-1, *A.* sp. GSX-BL, *A.* sp. KU2-6, and *A. tepidamans*), *Geobacillus* species (*G. caldoxylosilyticus*, *G.* sp. JF8, *G.* sp. NUB3621, *G.* sp. WCH70, *G. thermodenitrificans*, and *G. thermglucodisasius* NBRC 107763), and *Bacillus* species (*B. firmus*, *B. oceanisediminis*, and *B.* sp. 2 A 57 CT2). The conserved Asp-Glu-Asp catalytic triad residues are indicated by green triangles. The residues that coordinate Ca1, Ca2, Ca3, and Ca4 are indicated by red, green, blue, and purple stars, respectively.

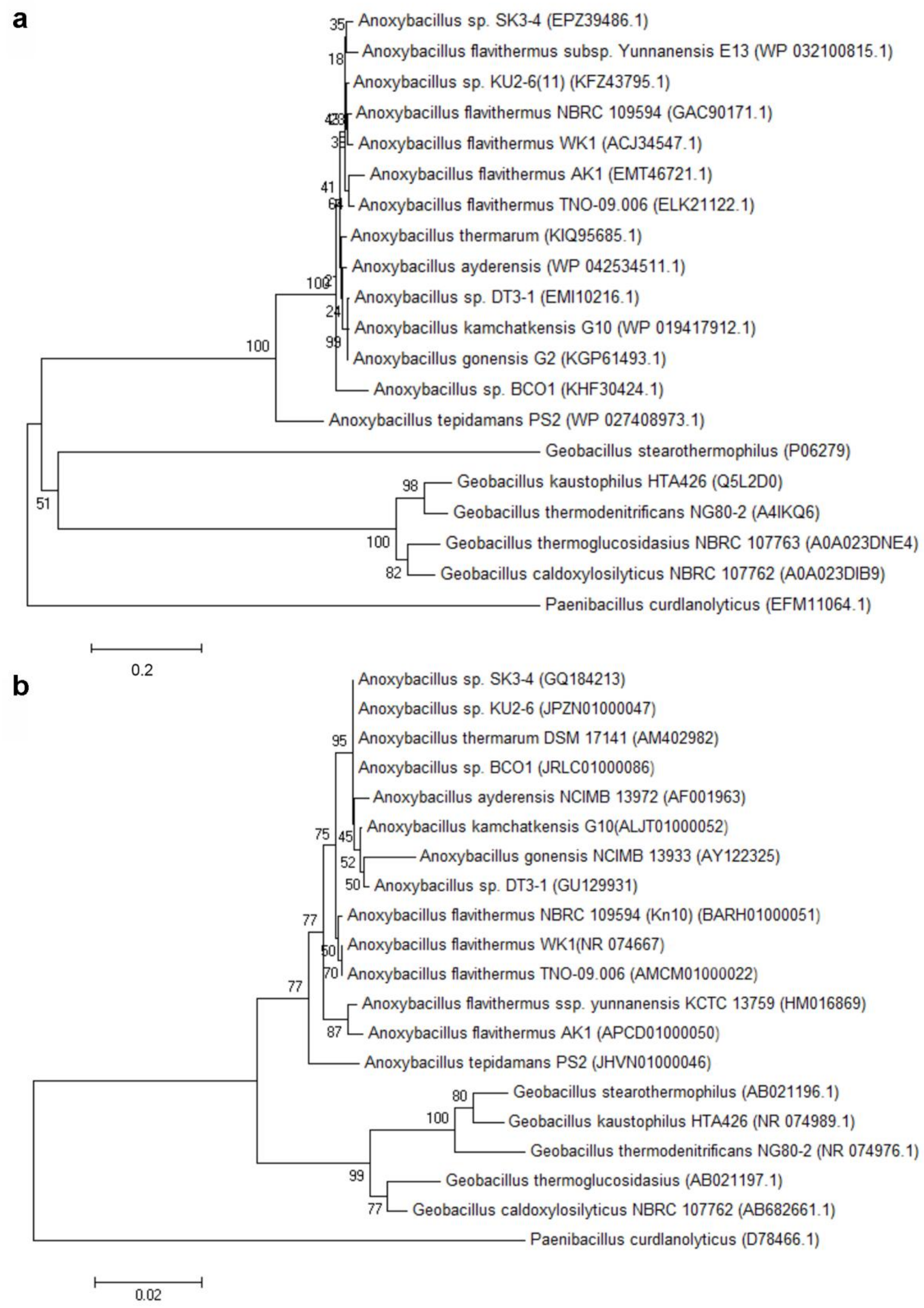

Fig. S2 Phylogenetic tree of *Anoxybacillus* α-amylases and 16S rRNA sequences with *Geobacillus* counterparts. (a) α-amylases. (b) 16S rRNA.

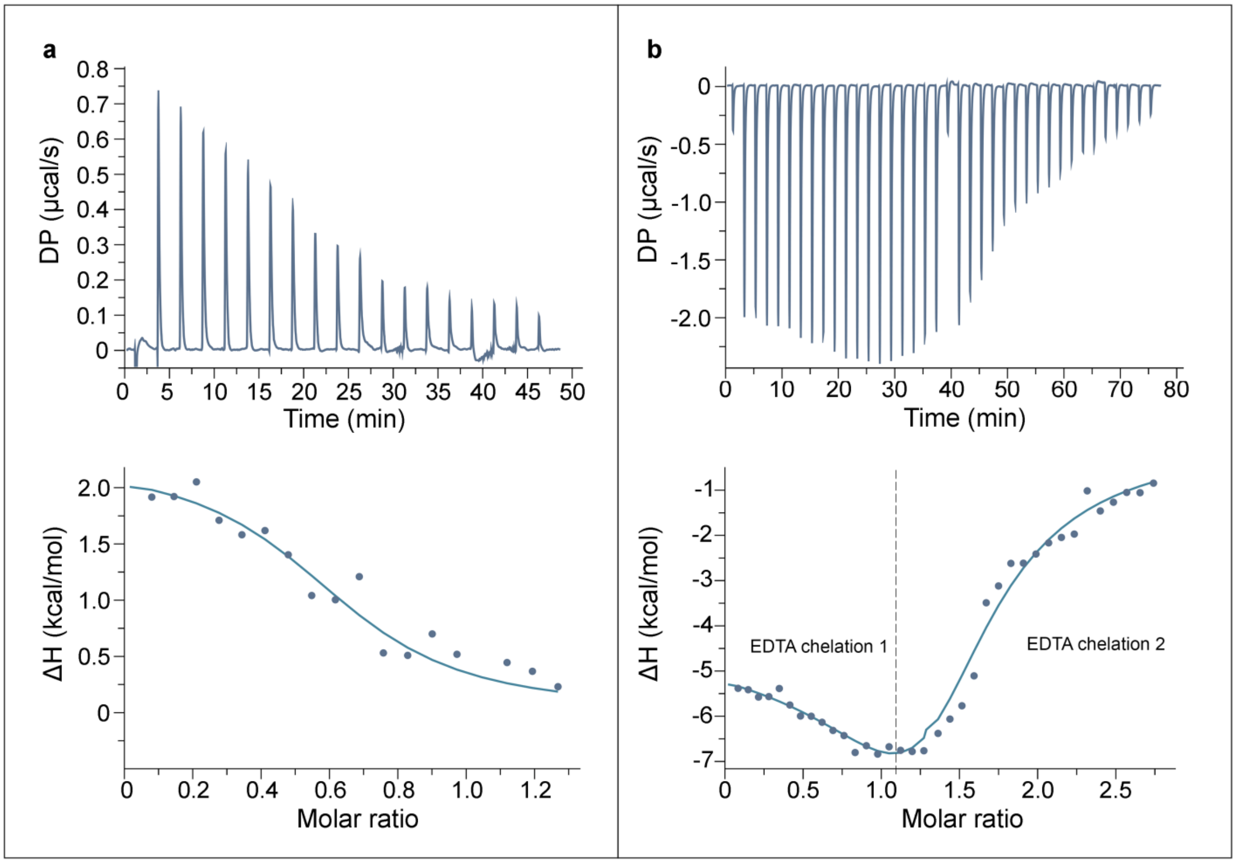

Fig. S3 Isothermal Titration Calorimetry of TASKA with (a) $CaCl_2$ and (b) EDTA. The upper panel of graph indicates heat flow of each injection (µcal/s) as a function of time (min); the lower panel indicates the integration of each peak (kcal/mol) as a function of molar ratio of TASKA and titrant. (a) At the end of titration, TASKA was saturated with Ca^{2+} ions. (b) The mixture of TASKA saturated with Ca^{2+} ions was then titrated with EDTA. Two EDTA chelation events were observed that the added EDTA first chelates the free Ca^{2+} ions in the mixture, then the TASKA-bound Ca^{2+} ions.

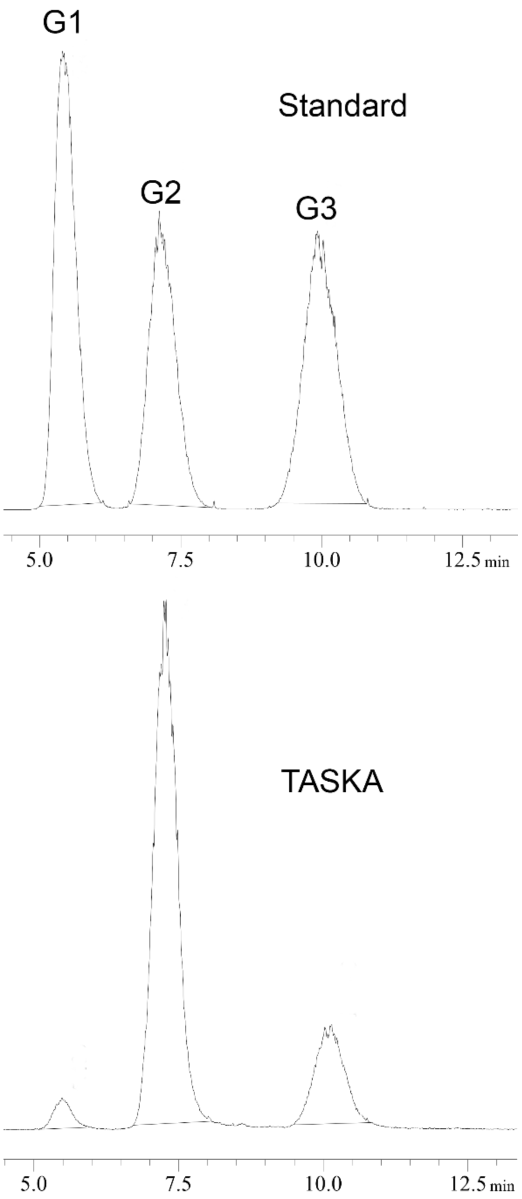

Fig. S4 UHPLC analysis of TASKA-catalyzed maltotriose hydrolysis. Chromatogram showing the maltotriose (G3) hydrolysed by TASKA producing glucose (G1) and maltose (G2).

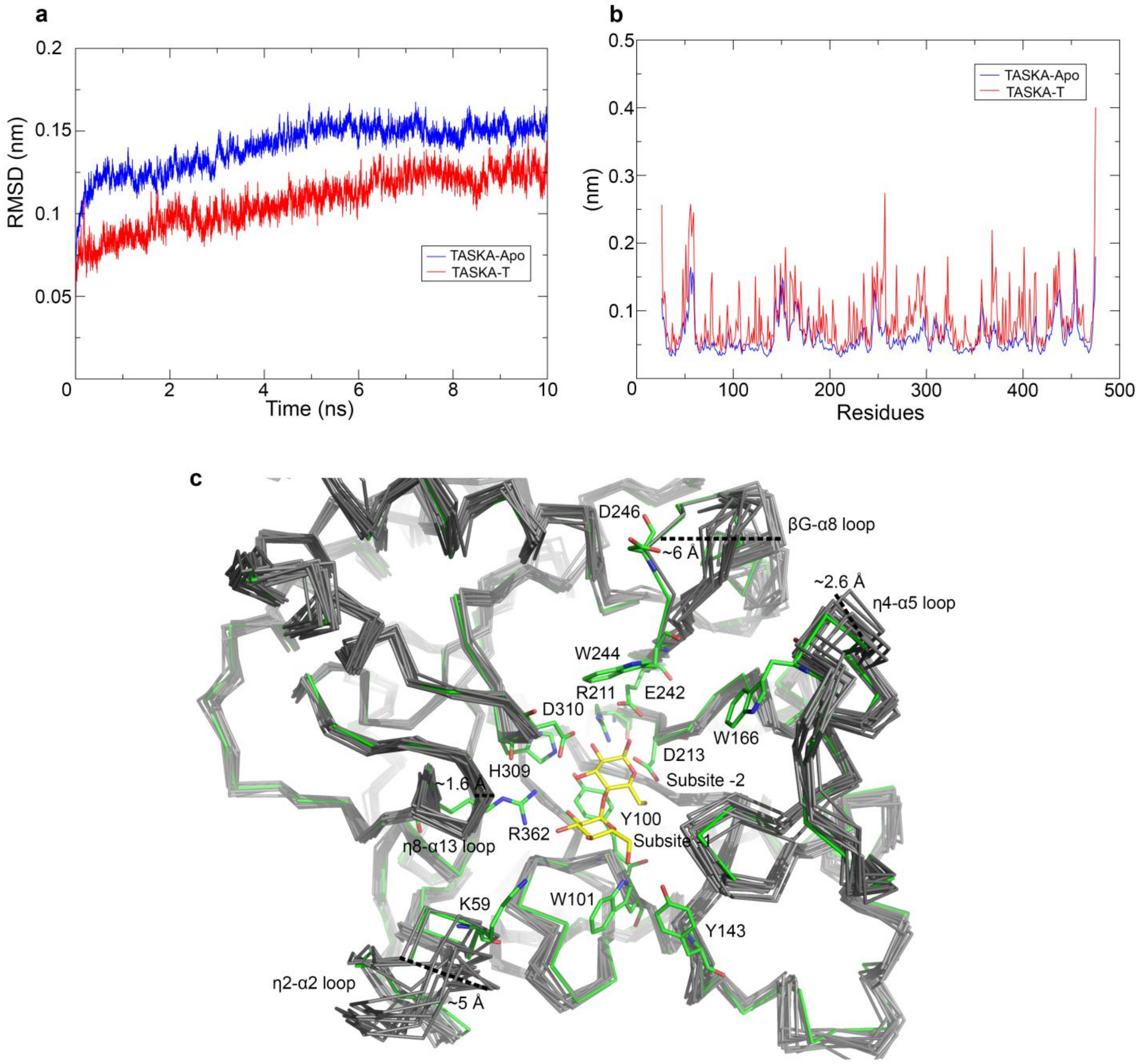

Fig. S5 Trajectory analysis of TASKA molecular dynamics simulation. (a) RMSD plot of TASKA-Apo (blue) and TASKA-T (red) at 293 K (b) RMSF plot of TASKA-Apo (blue) and TASKA-T (red) at 293 K. RMSF plot explained the residual and overall changes of TASKA. (c) Superposition of TASKA-T structures shows the flexible loops in proximity to maltose binding residues. The motion of the loop is represented by black dotted line.

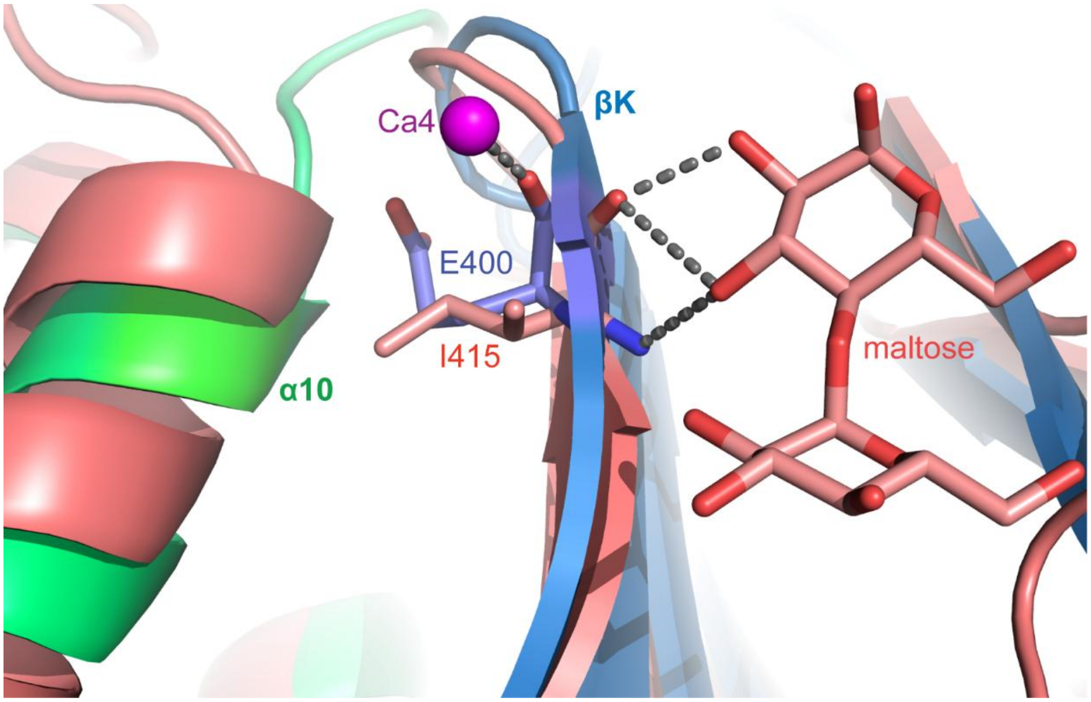

Fig. S6 Superposition of TASKA-Apo (domain C, blue; domain A, green) and *B. stearothermophilus* maltogenic α-amylase Novamyl (PDB ID: 1QHO) (salmon) shows the main-chain residue E400 of strand βK and the equivalent residue I415 of Novamyl that interact with maltose. Hydrogen bonds are shown as dotted lines.
